# Supplementary material for: Synergy in anti-malarial pre-erythrocytic and transmission-blocking antibodies is achieved by reducing parasite density
Source: eLife. 2018 Jun 19;7:e35213. doi: 10.7554/eLife.35213 (PMC6008048; doi:10.7554/eLife.35213)
Supplement: Transparent reporting form [file elife-35213-transrepform.pdf]

eLIFE
1st Floor
24 Hills Road
Cambridge CB2 1JP, UK
P 01223 855340
W elifesciences.org
T @elife

eLife's transparent reporting form

We encourage authors to provide detailed information *within their submission* to facilitate the interpretation and replication of experiments. Authors can upload supporting documentation to indicate the use of appropriate reporting guidelines for health-related research (see EQUATOR Network), life science research (see the BioSharing Information Resource), or the ARRIVE guidelines for reporting work involving animal research. Where applicable, authors should refer to any relevant reporting standards documents in this form.

If you have any questions, please consult our Journal Policies and/or contact us: editorial@elifesciences.org.

Sample-size estimation

- You should state whether an appropriate sample size was computed when the study was being designed
- You should state the statistical method of sample size computation and any required assumptions
- If no explicit power analysis was used, you should describe how you decided what sample (replicate) size (number) to use

Please outline where this information can be found within the submission (e.g., sections or figure legends), or explain why this information doesn't apply to your submission:

eLife Sciences Publications, Ltd is a limited liability non-profit non-stock corporation incorporated in the State of Delaware, USA, with company number 5030732, and is registered in the UK with company number FC030576 and branch number BR015634 at the address 1st Floor, 24 Hills Road, Cambridge CB2 1JP | August 2014

Preliminary work was carried out to estimate the appropriate doses required to statistically test for synergy between a transmission blocking and pre-erythrocytic antibody that can be upregulated by a vaccine. The experimental methodology has been published previously in Blagborough et al. (2013) which enables an effect size to be estimated for transmission-blocking drugs and vaccines. The statistical method has been published previously (Sherrrard-Smith et al. 2017) which enables a rigorous assessment of the effect size on parasite density as well as parasite prevalence.

Experiment sample sizes are noted below:

Stage 1 preparing for the appropriate TBV dose to use: (lines 178 - 203)
To titrate the appropriate dose, **5 female Tuck Ordinary (TO) mice** (6 – 8 weeks old, Harlan, UK) were treated with phenylhydrazine, and three days later, infected with 10^6 *P. berghei Pb*Pfs25DR3 (Goodman et al. 2011). Three days later, infected mice were injected intravenously (*i.v.*) with 200 µl of purified mAb-4B7 at a range of doses. Negative control mice were transfused with 200 µl of phosphate buffered saline (PBS). After 1 hour, mice were anaesthetised and **50 *Anopheles stephensi* mosquitoes** (line SD 500, previously starved for 24 hours) were allowed to feed on each individual mouse. Mosquitoes were maintained as described in (Blagborough et al. 2013), and after 10 days, **50 mosquitoes** were dissected and microscopically examined to measure oocyst intensity and prevalence. This was repeated five times, with *i.v.* administered doses of mAb-4B7 ranging from 0 µg to 750 µg. Anti-Pfs25 mAb 4B7 doses (via passive transfer) were titrated for 50%, 65% and 85% reduction in oocyst prevalence with PbPfs25DR3 via passive transfer and DFA.

Stage 2 preparing for the appropriate PEV dose to use: (lines 205 - 217)

An appropriate dose for mAb-3D11 was estimated from **40** individual passive transfers, administering a range of mAb-3D11 doses (0 – 150 µg i.v.) to mice (**5 to 10 mice** per experiment) and determining the prevalence efficacy at the given dose.

Stage 3 the experiment (following Blagborough et al. 2013) (lines 219 - 258)
Each group of mice (**5 mice** per group) either received the PEV antibody or no - intervention (negative control). Engorged mosquitoes were microscopically examined immediately after feeding to determine the number of sporozoites in the salivary glands. After 10 days, blood smears from each mouse were microscopically examined to determine the percentage parasitemia. These **5 mice** were then given either the TBV antibody at the desired dose to achieve a 50%, 65% or 85% reduction in oocyst prevalence as required, or no intervention/control (in accordance with the respective treatment arm). A new cohort of **500 naïve mosquitoes** was then allowed to blood feed on the mice. This mouse-to-mouse transmission cycle was repeated to **a maximum of 4 cycles after the seeding mouse population** or until no parasites had been detected in the system for 2 successive transmission cycles.

Blagborough, Andrew M, Thomas S Churcher, Leanna M Upton, Azra C Ghani, Peter W Gething, and Robert E Sinden. 2013. “Transmission-Blocking Interventions Eliminate Malaria from Laboratory Populations.” *Nature Communications* 4 (1812). Nature Publishing Group: 1812. doi:10.1038/ncomms2840.

Replicates

- You should report how often each experiment was performed
- You should include a definition of biological versus technical replication
- The data obtained should be provided and sufficient information should be provided to indicate the number of independent biological and/or technical replicates
- If you encountered any outliers, you should describe how these were handled
- Criteria for exclusion/inclusion of data should be clearly stated
- High-throughput sequence data should be uploaded before submission, with a private link for reviewers provided (these are available from both GEO and ArrayExpress)

Please outline where this information can be found within the submission (e.g., sections or figure legends), or explain why this information doesn't apply to your submission:

In the preliminary experiments to determine effect sizes, each experiment was repeated 5 times at appropriate doses (*lines 178- 217*).

The data for the main experiment are provided in Supplementary file – Table S1. All data were included in the model.

Statistical reporting

- Statistical analysis methods should be described and justified
- Raw data should be presented in figures whenever informative to do so (typically when N per group is less than 10)
- For each experiment, you should identify the statistical tests used, exact values of N, definitions of center, methods of multiple test correction, and dispersion and precision measures (e.g., mean, median, SD, SEM, confidence intervals; and, for the major substantive results, a measure of effect size (e.g., Pearson's r, Cohen's d)
- Report exact p-values wherever possible alongside the summary statistics and 95% confidence intervals. These should be reported for all key questions and not only when the p-value is less than 0.05.

Please outline where this information can be found within the submission (e.g., sections or figure legends), or explain why this information doesn't apply to your submission:

The statistical model was designed specifically for this experiment to look at both parasite density and prevalence and the stan code in the statistical software R is provided here: Sherrard-Smith et al. (2017). Occasionally mice were lost prior to exposure to mosquitoes, because the model was fitted to a global population, this could be accounted for because the model could be adjusted given the entire experimental data. All data were included in the model fitting and are provided in the supplementary information. (*lines 266 - 293*) The technical aspects of the model are provided in Sherrard-Smith et al. (2017). Figure 4 demonstrates the posterior draws from the Bayesian model to determine statistical support for synergy.

Sherrard-Smith, Ellie, Thomas S Churcher, Leanna M Upton, Katarzyna A Sala, Sara E Zakutansky, Hannah C Slater, Andrew M Blagborough, and Michael Betancourt. 2017. "A Novel Model Fitted to Multiple Life Stages of Malaria for Assessing Efficacy of Transmission-Blocking Interventions." *Malaria Journal*.

(For large datasets, or papers with a very large number of statistical tests, you may upload a single table file with tests, Ns, etc., with reference to sections in the manuscript.)

Group allocation

- Indicate how samples were allocated into experimental groups (in the case of clinical studies, please specify allocation to treatment method); if randomization was used, please also state if restricted randomization was applied
- Indicate if masking was used during group allocation, data collection and/or data analysis

Please outline where this information can be found within the submission (e.g., sections or figure legends), or explain why this information doesn't apply to your submission:

Data were allocated to a specific treatment and mosquito biting rate (1, 2, 5 or 10 mosquito bites per mouse).

This is explained in lines 229 -242

Pre-defined numbers of mosquitoes (to simulate mosquito biting rates of 1, 2, 5 and 10 mosquito bites per mouse) were … randomly selected from the … mosquitoes and fed, for 20 minutes, on anesthetized mice from a naïve cohort. The mosquito biting rate is an aspect of the experimental design that can be varied to be able to estimate the effect size more precisely. If the mosquito biting rate is small (say 1 or 2), the probability that the infection is eliminated rapidly in the intervention arm of the experiment is high (>80%) for TBD/TBV with efficacies above 40%. Thus, we cannot discriminate at a low mosquito biting rate between a TBD/TBV with 60% efficacy and one with 80% efficacy (they both eliminate). However, we do obtain a high degree of discrimination between an efficacy of 20% and one of 40%. Thus, a small mosquito biting rate is needed to get a precise estimate of the effect size of a TBD/TBV with lower efficacy. The converse also holds, so that a high mosquito biting rate (up to around 10 based on our initial experiment) is needed to obtain a precise estimate of a TBV/TBD with >80% efficacy. Using multiple mosquito biting rates increases the overall precision of our estimate of prevalence efficacy.

Additional data files ("source data")

- We encourage you to upload relevant additional data files, such as numerical data that are represented as a graph in a figure, or as a summary table
- Where provided, these should be in the most useful format, and they can be uploaded as "Source data" files linked to a main figure or table
- Include model definition files including the full list of parameters used
- Include code used for data analysis (e.g., R, MatLab)
- Avoid stating that data files are "available upon request"

Please indicate the figures or tables for which source data files have been provided:

All data are provided as an excel file in the Supplementary file – Table S1.
